# A rehabilitation intervention to promote physical recovery following intensive care: a detailed description of construct development, rationale and content together with proposed taxonomy to capture processes in a randomised controlled trial

**DOI:** 10.1186/1745-6215-15-38

**Published:** 2014-01-29

**Authors:** Pam Ramsay, Lisa G Salisbury, Judith L Merriweather, Guro Huby, Janice E Rattray, Alastair M Hull, Stephen J Brett, Simon J Mackenzie, Gordon D Murray, John F Forbes, Timothy Simon Walsh

**Affiliations:** 1 Lothian University Hospitals Division, 51 Little France Crescent, Old Dalkeith Road, Edinburgh EH16 4SA, UK; 2 Edinburgh Critical Care Research group, Edinburgh University and NHS Lothian, Chancellors Building, 49 Little France Crescent, Old Dalkeith Road, Edinburgh EH16 4SB, UK; 3 Interdisciplinary Social Science in Health/Nursing Studies, University of Edinburgh, Medical School, Teviot Place (Doorway 6), Edinburgh EH8 9AG, UK; 4 School of Nursing and Midwifery, University of Dundee, City Campus, 11 Airlie Place, Dundee DD1 4HJ, UK; 5 NHS Tayside, Perth Royal Infirmary, Perth PH1 1NX, UK; 6 Imperial College Healthcare NHS Trust, The Bays, South Wharf Road, St Mary’s Hospital, London W2 1NY, UK; 7 Centre for Population Health Sciences, University of Edinburgh, Medical School, Teviot Place, Edinburgh EH8 9AG, UK; 8 Centre for Inflammation Research, Queens Medical Research Institute, 47 Little France Crescent, Old Dalkeith Road, Edinburgh, EH16 4TJ, UK

**Keywords:** Critical care, Rehabilitation, Complex intervention

## Abstract

**Background:**

Increasing numbers of patients are surviving critical illness, but survival may be associated with a constellation of physical and psychological sequelae that can cause ongoing disability and reduced health-related quality of life. Limited evidence currently exists to guide the optimum structure, timing, and content of rehabilitation programmes. There is a need to both develop and evaluate interventions to support and expedite recovery during the post-ICU discharge period. This paper describes the construct development for a complex rehabilitation intervention intended to promote physical recovery following critical illness. The intervention is currently being evaluated in a randomised trial (ISRCTN09412438; funder Chief Scientists Office, Scotland).

**Methods:**

The intervention was developed using the Medical Research Council (MRC) framework for developing complex healthcare interventions. We ensured representation from a wide variety of stakeholders including content experts from multiple specialties, methodologists, and patient representation. The intervention construct was initially based on literature review, local observational and audit work, qualitative studies with ICU survivors, and brainstorming activities. Iterative refinement was aided by the publication of a National Institute for Health and Care Excellence guideline (No. 83), publicly available patient stories (Healthtalkonline), a stakeholder event in collaboration with the James Lind Alliance, and local piloting. Modelling and further work involved a feasibility trial and development of a novel generic rehabilitation assistant (GRA) role. Several rounds of external peer review during successive funding applications also contributed to development.

**Results:**

The final construct for the complex intervention involved a dedicated GRA trained to pre-defined competencies across multiple rehabilitation domains (physiotherapy, dietetics, occupational therapy, and speech/language therapy), with specific training in post-critical illness issues. The intervention was from ICU discharge to 3 months post-discharge, including inpatient and post-hospital discharge elements. Clear strategies to provide information to patients/families were included. A detailed taxonomy was developed to define and describe the processes undertaken, and capture them during the trial. The detailed process measure description, together with a range of patient, health service, and economic outcomes were successfully mapped on to the modified CONSORT recommendations for reporting non-pharmacologic trial interventions.

**Conclusions:**

The MRC complex intervention framework was an effective guide to developing a novel post-ICU rehabilitation intervention. Combining a clearly defined new healthcare role with a detailed taxonomy of process and activity enabled the intervention to be clearly described for the purpose of trial delivery and reporting. These data will be useful when interpreting the results of the randomised trial, will increase internal and external trial validity, and help others implement the intervention if the intervention proves clinically and cost effective.

## Background

Survival following critical illness can be associated with a constellation of physical, psychosocial and cognitive impairments that result in disability and reduced health-related quality of life (HRQoL) [1-4]. Prevalent issues include weakness, fatigue, breathlessness, poor nutritional state, anxiety, depression, post-traumatic reactions, and reduced cognitive function [1,5-9]. Many patients are unable to perform instrumental activities of daily living (ADLs), are slow or fail to return to employment, and require significant functional and psychosocial support from family members [10-13]. The economic implications of post-critical illness disability are poorly defined, but are likely to be high in terms of direct and indirect healthcare costs [14-16]. Observational studies have identified and quantified many aspects of post-critical illness disability, but there are few evaluations of rehabilitation interventions designed to support or expedite recovery. Published clinical trials suggest possible improvement in physical recovery from strategies that promote early mobilisation and muscle activity during intensive care [17-19], and the supervised use of a self-help manual in the early post-ICU period improved recovery of physical HRQoL scores [20]. In contrast, other strategies focussing on longer-term physical recovery through physiotherapy and exercise programmes, and follow up clinics, have not improved clinical outcomes [21-23].

Rehabilitation is a complex healthcare intervention, usually involving multiple components delivered by a range of healthcare professionals. Complexity is increased when the patients involved are heterogeneous in terms of impairment types, severity, concurrent comorbidity and when the trajectories of recovery vary. In addition, different impairments may interact in variable ways, which may affect the response to therapeutic interventions [24-26]. Studying post-critical illness rehabilitation is therefore highly complex. Failure of an intervention could occur because the components were ineffective, because they were not delivered as intended, or because other factors limited their impact (for example, psychological morbidity or patient inability to participate due to fatigue). It is also possible that improvements in important outcomes are missed if the measures used lack sensitivity or are measured at the wrong time [27].

The development and evaluation of complex healthcare interventions requires an alternative approach to that used for simple interventions such as novel drugs. The Medical Research Council (MRC) has published guidance on development, evaluation, and reporting, which recommends addressing the following questions when developing an intervention [27]:

•Are you clear about what you are trying to do, what outcome you are aiming for, and how you will bring about change?

•Does your intervention have a coherent theoretical basis which has been used to develop the intervention?

•Can you describe the intervention fully, so that it can be implemented properly for the purposes of your evaluation, and replicated by others?

•Does the existing evidence suggest that it is likely to be effective or cost effective?

•Can it be implemented in a research setting, and is it likely to be widely implementable if the results are favourable?

The extended CONSORT guidance for reporting non-pharmacologic randomised trials emphasises the need to fully describe interventions [28]. Specifically, rehabilitation trials should report the content of therapy sessions, how they were delivered, the information exchanged with participants, and the instruments used to provide information. Data describing the number, timing, and duration of each therapy session, its main component(s), and the overall duration of the intervention should be described, and it is recommended that the ways in which the intervention was tailored to individual patient needs is reported. Wells and colleagues [29] emphasised the importance of describing and understanding context in complex intervention trials to maximise understanding of the external relevance of findings outwith trial settings.

We developed a complex healthcare intervention to promote physical recovery following critical illness, which is currently being evaluated in a randomised trial: the RECOVER study (ISRCTN09412438). The trial protocol has been published [30]. The aim of this paper is to describe how the RECOVER intervention was developed and present a taxonomy for describing the treatments delivered. The information included is intended to enable other clinicians, researchers, and healthcare providers to fully understand the rationale for the intervention. We anticipate this will improve the external validity of the trial findings by providing a level of information that will help interpret the findings. The detail provided in this paper will aid reproduction of the intervention in other settings.

## Methods

The intervention evolved between 2005 and 2010. The multidisciplinary research group included an academic critical care physician (TSW), two academic ICU nurses (PR, JER), an academic physiotherapist (LGS), a clinically and research active dietitian (JLM), a psychiatrist with an interest in post-critical care psychological morbidity (AMH) and a critical care physician in a senior National Health Service (NHS) management role (SJM). Methodologists included a social anthropologist with expertise in health service change (GH), a trialist/statisticians (GDM, Steff Lewis (MRC methodology hub; Edinburgh University) and a health economist (John Forbes (Centre for Population Health Sciences, Edinburgh University)).

The optimum methodology for developing rehabilitation trials for patients experiencing critical illness has not been determined, and we did not define this *a priori* at the start of the research programme. The process used was iterative and a range of factors influenced the final construct, including responses to reviewers during sequential revisions of grant applications. Individual and collective brainstorming strongly influenced the final intervention, which was also influenced by emerging local data from collaborators undertaking post-graduate degrees and post-doctoral work during this period [31-33]. These individuals adopted lead roles in defining specific elements of the intervention, but group activities ensured all ideas were heard.

Inputs from patients and carers were collated from several sources: first, a local qualitative study with survivors of prolonged mechanical ventilation [31]; second, discussions with former patients during a “pilot” post-ICU service (see below); third, a range of interviews publicly available at Healthtalkonline (http://healthtalkonline.org); fourth, a James Lind Alliance event organised by the Edinburgh Critical Care Research Group at which the perceived needs of patients during the post-ICU period was discussed [34]; and fifth, a patient representative on the trial steering group, who had experienced a prolonged recovery following severe acute respiratory distress syndrome.

During the iterative development process it became clear that defining the content of the intervention, its timing in relation to the recovery process, and the method of delivering it were of central importance to both trial design and subsequent interpretation of findings. These are each considered separately. Following agreement of the final construct, a taxonomy was developed to fully describe the intervention components, and facilitate adequate reporting during the trial.

## Results

### Defining components of the intervention

The range of research, feasibility, and piloting activities that contributed to defining the intervention content are summarised below.

#### Literature review

We undertook a detailed literature review, which indicated consistent reports of physical disability after ICU discharge, which was most marked in the initial months and tended to recover with variable trajectories over 3 to 6 months [1,35,36]. Many patients did not regain pre-illness function. It was also relevant that many patients report pre-existing comorbidity and physical impairment [37-40]. Poor appetite and altered taste were widely reported, which could contribute to slow nutritional recovery and/or failure to regain pre-illness weight [5]. The published literature suggested these features are most prominent during the initial weeks and months following ICU discharge, corresponding to the period in hospital and early after discharge to community/primary care. However, the duration of symptoms and disability clearly vary widely between individuals in type, severity and duration, and no validated method for predicting individual recovery trajectories was found.

In addition to physical disability, a high prevalence of psychological morbidity following critical illness was reported [6,7,9,36,41-43]. This included anxiety, depression, and post-traumatic stress symptomatology with widely ranging prevalence depending on case mix, time of measurement, and measurement tool [43]. Emerging evidence over the development period suggested symptomatology was increased by pre-illness factors (for example, previous psychiatric history) [6,41], a mixture of non-modifiable and potentially modifiable ICU related factors (greater illness severity, delusional memories, delirium, over-sedation) [41,44], and potentially modifiable post-ICU factors (provision of information, patient diaries) [45,46]. In addition to psychological morbidity, the literature indicated significant cognitive decline in many patients following critical illness, including impaired memory, executive function [9,47,48] and limitation in instrumental ADLs [38,47]. There were few published trials of therapeutic interventions over the course of intervention development. Many of these were restricted to the time in ICU. Several studies strongly influenced the development of our intervention (Table 1).

**Table 1:** Rehabilitation trials after critical illness that strongly influenced the intervention development

**Trial**	**Summary**
Jones *et al*. 2003 [20]	Ward-based: Self-help manual over 6 weeks, which improved physical function (measured using the SF-36 PCS) at 2 and 6 months following ICU discharge. Patients were recruited to the study within 1 week of discharge from intensive care. The intervention group received the self-help manual in addition to the routine ICU follow-up that all patients received.
Schweickert *et al*. 2009 [49]	Intensive Care: Early exercise and mobilisation (physical and occupational therapy) during sedation breaks in intensive care. Patients were recruited if less than 72 hours of mechanical ventilation. Return to independent functional status at hospital discharge was significantly higher in the intervention group. They also had a shorter duration of delirium and more ventilator-free days compared to the standard care group. The intervention was delivered primarily in the ICU.
Cuthbertson *et al*. 2009 (PRaCTICaL) [21]	Discharge Home: Nurse-led follow-up clinic at 3 and 9 months post-hospital discharge, in combination with a manual based, self-directed, physical rehabilitation programme introduced in hospital. At 12 months, there were no statistically significant differences in HRQoL or any secondary measures between groups.
HRQoL, health-related quality of life; SF-36 PCS, SF-36 Physical Component Score	

During intervention development, the National Institute for Health and Care Excellence (NICE) published a short guideline for rehabilitation after critical illness [36]. This included a formal systematic review and recommendations based substantially on the multidisciplinary expertise of the guideline development group (GDG). The systematic review did not identify important new evidence compared to our literature review. The GDG recommended sequential assessments at key points in the patient pathway, with provision of physical and psychological rehabilitation when appropriate. The GDG acknowledged, however, that the most appropriate assessment tools and rehabilitation interventions were unknown. A summary of the key recommendations is shown in Table 2. To ensure our intervention was consistent with the recommendations and expertise of the GDG we invited the Chair (SJB) to act as independent Chair to the RECOVER Trial Steering Committee, offering advice and additional expert review.

**Table 2:** Summary of recommendations from National Institute for Health and Care Excellence clinical guideline 83

**General recommendations**	**Rehabilitation should be undertaken by individuals with relevant skills and knowledge communication between teams and professionals over the rehabilitation pathway should occur**
**Pathway-specific recommendations**	
During critical care stay	Assess risk of post-ICU disability
	Commence goal-oriented rehabilitation early
	Involve families and carers
	Provide illness-related information to patient and family
	Optimise provision of nutrition
At ICU discharge	Screen patient for physical and psychological issues
	Plan individualised rehabilitation programme with defined goals
	Provide information to patients and families about rehabilitation pathway, likely morbidity, ICU stay, and transition to general ward environment
During ward based care	Repeat screening for physical and psychological issues
	Offer individualised rehabilitation programme with defined goals, provided by a multidisciplinary team
	Regularly update rehabilitation programme and goals, making specialist referral where appropriate
	Offer structured self-directed and supported rehabilitation manual for at least 6 weeks to appropriate patients
Prior to hospital discharge	Perform a functional assessment including physical and psychological elements, evaluating the impact on patient activities of daily living and participation
	Ensure support for outstanding issues are arranged, including ongoing rehabilitation by community services
	Provide patient and family with relevant information, including information about their ICU stay
At 2–3 months post-ICU discharge	Review patient as outpatient and perform functional assessment
	Refer for ongoing rehabilitation and/or specialist support according to individual need
Adapted from [36]	

#### Work with patients and families

Qualitative interviews with former patients [31] indicated a range of important issues during the weeks following ICU discharge that were important to patients. Thematic analysis resulted in key issues to incorporate in the trial intervention (Table 3). Some were generic (for example, coordination and continuity of care, preparation for discharge home, providing information) while others related to specific issues (for example, physiotherapy provision, individualised therapy, discussion of memories). Many of these were supported by existing literature, and were also reported by patients and families during discussions at the James Lind Alliance event [34].

**Table 3:** Key themes from interviews with survivors of critical illness

**Theme**	**Illustrative quotes**
**Coming to terms with memories and experience of ICU**	“I was convinced that Jack (the Ripper) was going to…slit my throat, that he’d killed 2 nurses and he’d dumped their bodies in a bin down the side of the stairs. It really was frightening”.
**Needing knowledge and information**	“…it all just suddenly clicked into place…it suddenly became a hospital. I suppose I was…getting the drugs out of my system. Certainly, those first days, I was in the twilight zone…”
• Waking up and not knowing what has happened	“I said, “Tell me once I’m better. Don’t tell me just now, because every day is a battle”. I really didn’t want to hear…how close to death I’d been”.
• Reliance on family for informational needs and the need for flexibility in terms of timing	“Even in my fuzzed head, I was aware on a number of occasions that whoever was momentarily in charge of me had scant knowledge of who I was and how I got there”.
• Poor continuity of care/inability of ward-based staff to provide information on the critical illness event	
**Dealing with physical disability**	“I don’t know if it’s something that happens if you’ve only been in [ICU] a few days…but your body feeds off your muscles. I didn’t know any of this…Had I have had this knowledge, it would’ve been…easier for me to accept”.
• Making sense of functional impairment and dependence	“I was told I’d get very intensive physio…and then I got none for 5 days straight. It was only when I made a fuss that I got it. And then I got…just a list of things to do on my own…that were way beyond my capabilities”.
• Frustration with brevity, frequency, delivery of physical therapy in relation to perceived needs	“I’d get maybe 10 minutes of physiotherapy every day. Eventually. It wasn’t particularly aggressive physiotherapy…being hoisted up in a stand aid, and sitting down again. In terms of getting you back on your feet, it was minimal”.
• Regaining functional independence as priority	“I was determined I was gonna get mobile as quick as possible. I’ve got that determination. I’ve had it all my life”.
• Feeling outside the rehabilitative process	“I had to fight with them at first, but then they let me do things at my own pace. I said to them “I will walk and I will do this, but you’ve got to let me do it…my own way”.
**General ward staff awareness**	“I had to get some assistance having a seated shower. I couldn’t stand because I was so weak…and they maybe showed a bit of impatience with me there”.
• Perceived insensitivity of staff to limitations and basic care needs	“I said, “I never should’ve been left the way I was. I should’ve done exercises so that I wasn’t in this state.” And Dr Charmless said to me, “Well, that can’t be helped”.
• Lack of understanding of their limitations and its cause (ICU-acquired weakness)	
**Hospital discharge planning**	“When I first got home, I got the shock of my life…I could put water in the kettle, but I couldn’t lift it. That’s when you say to yourself, “You *are* bad”.
• Pressure on beds; patients often discharged with limited functional ability	“I’m still waiting (for a bath seat), and I don’t know whether to ring back or persevere. Maybe somebody’s need is greater than mine. But initially, it would’ve been a big help”.
• Poor communication between acute and community teams; lack of timely provision of home aids	
**Early life at home**	“I was glad to be home but very, very tired and very weak. I had to rely on someone to help me get up, dress me, that sort of thing”.
• Not being adequately prepared for dependence on others	“I could’ve done more…to help myself…because my brother asked for a sheet of exercises for me to do when I got out. I realise now…I could’ve been doing a lot of that…and I think I could’ve progressed quicker”.
• Lack of guidance in terms of self-management of the recovery process	“…one afternoon, I walked right over there (gestures out of the window). But I was so knackered later that day that I daren’t go out the next day at all. At first I thought, “Oh, I’ll perhaps do this every day”, but I’ve not been out since (laughs)”.

#### Audit of existing service provision

To provide additional local data, we prospectively audited current provision of rehabilitation between ICU and hospital discharge in one institution (Royal Infirmary of Edinburgh, Scotland), with a focus on the level of input in terms of physiotherapy, dietetic, and other allied health services [32]. We noted that physiotherapy sessions were of low frequency and intensity and limited range. Dietetic management was limited to review of nutritional status and requirements, and advice about artificial nutrition or provision of supplements. Follow-up to explore whether nutrition was improved (for example, through food diaries, monitoring intake of supplements, assisting with eating, or individualisation of food provision) was rarely achieved. Systematic use of referral triggers to rehabilitation specialists such as occupational therapy and speech and language therapy was absent. Those referrals which did occur did so in an *ad hoc* manner. Many patients were not assessed or reviewed by these specialists until close to hospital discharge. Important general observations were that patients discharged from critical care became widely dispersed across the general wards, usually according to their “parent” specialist team. This resulted in a dilution of knowledge and expertise among medical, nursing and allied health professional staff of their history and problems, which affected continuity of care. A striking observation was that large numbers of different healthcare professionals reviewed patients at different times often in an uncoordinated manner [33]. Few professionals had time to gain an understanding of complex histories and clinical course of patients. In addition, patients effectively “competed” with less severely unwell patients (for example, elective surgery or short-term medical admissions) for scarce rehabilitation resource. There was an impression that shortages of ward beds potentially resulted in prioritisation of less sick patients for rehabilitative provision because they were more likely to be discharged quickly. Post-ICU patients were usually discharged directly home, were rarely offered formal rehabilitation, and when this occurred there was usually a long delay in transfer. Hospital discharge planning was poorly coordinated and the information provided to general practitioners and other community staff was inconsistent and often incomplete.

#### Pilot provision of post-ICU rehabilitation multidisciplinary ward round

Two ICU consultants (including TSW) piloted a multidisciplinary weekly round of patients who spent >4 days in the ICU with members of the research team representing a nursing (PR), dietetic (JLM), and physiotherapy (LGS) perspective. A log was kept to capture key contributions that might be incorporated in the trial intervention. Several additional issues emerged from this work: first, patients and families had limited access to information about the ICU stay or likely recovery trajectory from medical, nursing or other staff; second, review of medical notes often revealed medication changes that were overlooked (for example, stopping stress ulcer prophylaxis, antibiotic review, re-starting usual medications); third, patients were frequently socially isolated (often placed in a side room) because of real or perceived infection risk (for example, MRSA colonisation) or debility; fourth, patients frequently had prominent traumatic delusional memories that were not being aired, they did not understand, and were often only revealed when questioned sensitively; fifth, the information provided to community teams was often medicalised and not relevant to the rehabilitation needs of the patient; sixth, the demands and workload of the multidisciplinary team on general wards, especially nursing, dietetic, physiotherapy, and medical staff, meant they rarely met together to coordinate case management of complex post-ICU patients; finally, decisions about hospital discharge were often determined by medical staff from the ‘parent speciality’ team without multidisciplinary planning or negotiation with individual patients and/or their family members.

#### Feasibility trial to deliver enhanced physical and dietetic therapy with a generic rehabilitation assistant

A single-centre feasibility trial was undertaken to explore whether a GRA could work effectively under the supervision of multiple existing specialist teams to improve access to rehabilitation, coordinate case management, and deliver therapy. The results of this trial have been published, together with an individual case study [32,33]. This study established a model for a GRA with multiple skills working across several teams (primarily physiotherapy) under senior supervision. It also enabled the educational requirements of the role to be identified and developed, competencies defined, and the development of trigger tools for referral to senior specialists. The study specifically highlighted the need for pacing and individualised patient-centred goal setting techniques for both dietetic and physiotherapy rehabilitation. The high levels of fatigue immediately following intensive care discharge and wide variation in ability between patients highlighted the need for individually tailored rehabilitation programmes.

### Selection of key intervention elements

Based on the above, we decided to focus our intervention primarily on physical rehabilitation, because physical impairments were highly prevalent, appeared to strongly influence length of acute hospital stay, and profoundly affected the lives/ADLs and HRQoL of patients. The intervention components focussed primarily on better coordination and increased delivery of physiotherapy and nutritional rehabilitation, with a clear strategy to engage occupational therapy and speech and language therapy when required. An emphasis was placed on individualised rehabilitation to suit patient ability, with the intention of including patient-centred goal setting to the rehabilitation process. The only post-ICU intervention with evidence of effectiveness from clinical trials was the use of a self-help manual [20], so we chose to provide these to all participants, irrespective of group allocation in the trial. Our intention was for the standard care group to receive these in the context of existing rehabilitation services, whereas the intervention group would receive them as part of their enhanced rehabilitation.

Physical rehabilitation was supplemented by information giving in the form of a consultant visit, lay summary, and the offer of an ICU visit. These interventions focussed on explanation of events during ICU admission, current symptomatology (including delusional memories), and what to expect during recovery.

We recognised the importance of psychological morbidity to recovery. From the content experts in our group (AMH and JER), it was clear that the progression and evolution of psychological symptomatology is difficult to predict, and that evidence for the optimum intervention, its timing, and efficacy are very uncertain during the early recovery period. We therefore chose to provide enhanced information provision as part of the intervention, educate staff in the principles of post-ICU psychological problems, but not include a formal psychological intervention.

### Timing of the intervention

We decided to focus our intervention from the time of ICU discharge for up to 3 months, when the primary outcome of physical function was measured. Evidence for benefit from early physical mobilisation during mechanical ventilation emerged during the intervention development [49-51], and was being progressively implemented as a standard of care in the participating ICUs. In addition, the NICE guidance made research recommendations to evaluate the impact of coordinated rehabilitation, and the impact of specific therapeutic components, at key stages of recovery [36]. We decided to exclude the ICU stay, because this treatment would be difficult to control and the focus of our intervention was multidisciplinary rehabilitation during the later stages of recovery. In the UK, where the trial was planned, most patients are discharged to general ward care within a short time of discontinuing mechanical ventilation and other organ support (typically 1 to 2 days). On a practical level, recruiting patients earlier during mechanical ventilation introduced the competing risk of death as a potential major problem with trial design, because hospital mortality for patients requiring >48 hours of mechanical ventilation is typically 20 to 25% [52]. This was potentially problematic where the proposed primary and important secondary outcomes were measures of disability and HRQoL. Pragmatically, we thought the complexity of delivering and describing a multidiscipinary complex intervention during and after ICU, two environments with very different implementation challenges, would increase the risk of unsuccessful project completion. The entry point was therefore the time a patient was deemed fit for discharge from the ICU by the responsible physician.

**Figure 1:**
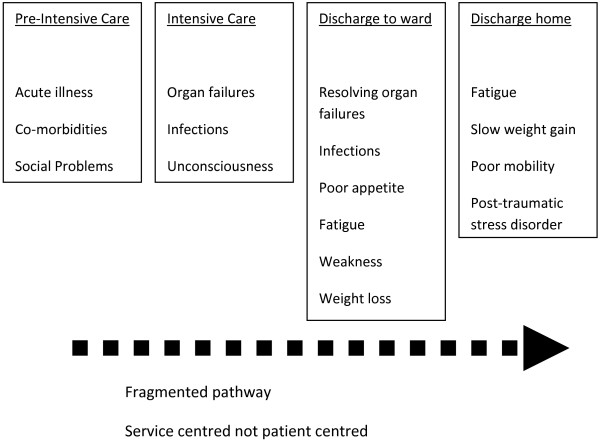
Critical illness pathway with key stages of recovery and transitions of care.

We chose to end the period of intervention at 3 months post-randomisation irrespective of patient status or location. Our rationale was that the steepest trajectory of physical recovery occurs during this period, but the literature clearly indicates persisting disability among many patients at this time point [1,3,4,53]. We expected this intervention period to include the transition to general ward care for all patients, the full period of general ward care for the majority of patients, the transition to post-acute hospital destination for the majority of patients, and a period of living at home or other post-discharge placement for most patients (Figure 1). As such, our intervention period included the key areas of need identified during development; it was clearly defined for the purpose of trial design and reporting. Previous studies have used hospital discharge as the outcome assessment point, which is influenced by factors other than disability, and is potentially subject to researcher bias in non-blinded trials [49,51]. We chose not to include clinical review at 2 to 3 months as part of the intervention. This decision was influenced by the negative results of a high-quality trial of ICU follow-up clinics [21], the limitations of what could be achieved with available resources, and the pre-trial qualitative and audit data indicating the high unmet needs during the first 3 months following ICU discharge.

### Method for delivering the intervention

We decided to employ dedicated GRAs as the key structure underpinning our intervention, extending the role from our feasibility trial [32]. We used the feasibility trial and audit data to anticipate the number of GRAs required across the two participating hospitals (2.5 whole-time equivalents; three individuals). The salaries for these individuals was identified as an additional treatment cost for the purpose of economic evaluation, and specific appointments to the posts were made by interview using an agreed job specification (available on request). We developed a clear educational content and competencies across physiotherapy, dietetics, occupational therapy, and speech/language therapy. The GRAs were trained according to these prior to starting the trial during a 1-month programme that included general education about critical illness and recognition of psychological issues after intensive care. A description of the training programme is included in Additional file 1. We developed a comprehensive competency document covering generic and discipline-specific skills required by the GRAs for this post. Individual competencies were assessed by qualified health professionals from the relevant disciplines. GRAs were given the opportunity to observe new skills and undertake them with supervision before they were deemed competent. This process is now being formalised further with the adoption of the Calderdale Framework [54]. We planned to embed these individuals in existing NHS teams, but limited their clinical activity to patients randomised to the intervention group. This was important to protect time to ensure delivery of the intervention as intended, and also minimised or eliminated various form of bias that are problematic in rehabilitation studies, including crossover effects, performance bias, differential expertise bias, instruction bias, and bias in using screening tools [28]. It also reduced but could not eliminate the impact of learning and experience during the course of the trial.

### Final construct and taxonomy

The final construct was agreed by a process of consensus, iteratively incorporating all of the data sources described above as they became available. The final peer review was by the Trial Steering Committee prior to submitting the final protocol for approval. Importantly, this included the independent Chair (SJB, a critical care clinician and chair of the NICE 83 GDG), a patient representative (a survivor of prolonged critical illness), and two independent content experts (a consultant and clinical manager of a medicine of the elderly service; and a consultant in rehabilitation medicine). In defining the final construct, we established a clear taxonomy to describe what was intended to occur, aiming to use this to structure the collection of process data during the trial. Wherever possible, we incorporated recommendations made in the MRC [27] and modified CONSORT guidance [28], anticipating that reporting would clearly describe what was done during the trial for both intervention and usual care groups. The parts of the patient pathway targeted by our final construct were discharge to the ward, during the acute hospital ward phase, at discharge from acute hospital, and after discharge home via telephone support (Figure 1). These included three of the five key stages identified in NICE guidance, namely ICU discharge, during ward-based care, and at discharge home (excluding during intensive care stay and formal follow-up at 2 to 3 months following hospital discharge).

The key elements of our complex intervention, their timing, frequency, healthcare workers involved, training of individuals delivering the intervention, fixed versus flexible elements, and degree of participation required from patients for successful delivery are shown in Table 4. This structure was adapted from suggestions by Wells and colleagues [29] to maximise information about the dimensions and context of the complex intervention. Adequate description of how the intervention was actually implemented and received by patients was expected to be vital to the internal and external validity of the findings, and interpretation of results. We incorporated processes that were identifiable, quantifiable and directly comparable into a process evaluation, which was included in the trial analysis plan. Key trial materials that operationalized the intervention, including the GRA education programme, the trigger tools used by GRAs for senior referral, the topic guides used for the consultant meeting, examples of the lay summary, the patient-centred goal-setting sheet and the discharge information letter sent to general practitioners by the GRAs are all included in Additional file 1. The trial analysis plan (which included the process evaluation plan) is available with the published protocol [30].

**Table 4:** **Detailed description of the rehabilitation construct adapted from recommendations from wells and colleagues**[29]**, the Medical Research Council framework**[27]**, and the modified CONSORT guidance for randomised controlled trials of non-pharmacologic randomised controlled trials**[28]

**Stage of patient pathway**	**Component of intervention**	**purpose**	**Structure and components**	**Theory/rationale**	**Flexibility to individual patient**	**Degree of active patient participation required**	**Healthcare professionals involved**	**Attributes and relevant training of healthcare professional**	**Location**	**Timing**
**Stage one – ICU discharge**	Introduction of patient to GRA, initial assessment, and explanation of rehabilitation strategy	To establish relationship between GRA and patient	Initial meeting	Early commitment to provide individualised rehabilitation and information will promote engagement, trust and reassurance, and reduce perception of abandonment	Low	Low	GRAs together with existing rehabilitation teams (primarily physiotherapy and dietetics)	GRA competency-based training in assessment and awareness of common ICU problems	General ward; occasionally in the ICU prior to discharge	As soon as feasible following allocation to intervention group
		Provide information to patient and carer	Formal assessment of function		Timing to suit patient, but within 1 day of randomisation in most cases					
			Setting initial rehabilitation goals							
	Meeting between patient and ICU consultant, with involvement of GRA and family where appropriate	To provide information about ICU stay and likely problems during recovery	Scheduled meeting	Information will reduce stress and anxiety	Moderate	Moderate	ICU consultant.	ICU consultant familiar with topic guide, knowledge of generic post-ICU issues and the individual patient history	General ward	During the first week or when deemed most appropriate by the GRA
		Opportunity for patients and family to ask questions	Topic guided discussion to cover physical and psychological sequelae of critical illness^1^	Filling in gaps and exploring delusional memories may reduce psychological morbidity	Optional; patient may decline meeting		Meeting usually attended by GRA			
				Answering questions and providing realistic expectations may help adjustment	Meeting tailored to individual patient and family					
	Provision of lay summary of ICU stay	Provide information about ICU stay and likely problems during recovery in understandable format	Lay summary dictated by consultant familiar with patient history using standard proforma^2^	Information in summary will achieve similar outcomes to the consultant visit and/or consolidate information given	Moderate	Low	ICU consultant to generate summary	ICU consultant familiar with topic guide, knowledge of generic post-ICU issues and the individual patient history	General ward	During post-ICU hospital stay
				Written summary can be used as ongoing resource by patient and family	All patients provided with summary, but decision regarding how and when to read this and use it at patient discretion		GRA to provide it to the patient, often with additional explanation	GRA with relevant training to assist patient in understanding content if needed		Posted to patient post-discharge if not available prior to hospital discharge
				Important as poor memory and other cognitive impairments may limit retention of information from meeting						
	Provision of self-help rehabilitation manual	Provide a resource to support recovery process	Manual that improved physical recovery in a previous randomised controlled trial^3^	Supported use of the self-help manual improved physical function components of quality of life questionnaires when used during the first 2 months following ICU discharge	Moderate	High	GRA	GRA familiar with the content and goals of the manual	General ward	Early during the post-ICU stay
					Manual provided to all patients					
					Use tailored to individual patients					
**Stage two –Ward-based rehabilitation**	Regular assessment by GRA	To assess patients using a combination of clinical judgement and standardised screening tools in relation to:	Frequent assessment and reassessment	A regular structured approach by a single individual to identify problems across multiple areas that potentially contribute to disability will improve coordination of care by senior rehabilitation staff who often working separately	Moderate	Moderate	GRA	GRA trained to defined competencies in each area	General ward	Throughout acute hospital stay
		Physical function. Nutritional status and dietary intake	Use of screening tools to trigger specialist advice from: physiotherapy, dietetics, occupational therapy, and speech and language therapy^4^	Consistency across multiple relevant areas will reduce the chance of one unaddressed issue slowing overall recovery	Frequency and timing of formal assessments and use of screening tools at discretion of GRA, but expected to occur weekly	Screening and assessment largely undertaken by GRA		GRA trained in use of screening tools		
		Activities of daily living			Informal assessment on more frequent basis					
		Communication and swallowing								
	Individualised goal setting	To set achievable realistic rehabilitation goals, individualised to each patient	Documented individualised goals agreed between rehabilitation team and patient	Individualised goal-setting is effective in other rehabilitation settings	High	High	GRAs and senior specialist rehabilitation staff as necessary	Training in the use of goal-setting in rehabilitation settings	General ward	Throughout acute hospital stay
			Regularly revised	Allows patient to focus on issues important to them	Intention to define achievable goals approximately weekly, but adjusted to individual patients				Potentially other settings (home visits; trips to other areas)	
				Patient feels empowered and involved						
				Achieving goals and documenting progress may have additional beneficial effects on psychological morbidity						
	Therapy sessions	Provide therapy sessions designed to achieve rehabilitation goals	Individually tailored therapy in areas of: Physiotherapy	Physiotherapy will improve the prominent symptomatology, and restore abilities to undertake ADL	High	High	GRA	Competency-based training in all relevant areas	General ward	Throughout acute hospital stay
			Dietetics	Dietetic therapy will address weight loss and barriers to nutritional recovery, such as poor appetite		Discrepancy between intended therapy and treatment achieved by patient strongly influenced by patient fatigue, mood, delirium, and many other issues	Planning and advice from senior rehabilitation specialists		Physiotherapy department	Timing and frequency determined by GRA and rehabilitation teams. Target at least one session per day from GRA monday to friday
			Occupational therapy	Occupational therapy will restore ability to undertake ADLs, reduce disability, and improve independence			Variable amount of therapy provided by senior specialists according to individual need		Occupational therapy department	
			Speech and language therapy	Speech and language therapy will treat specific swallowing problems or communication issues					Other hospital areas (stairs and mobility)	
			Pre-defined sub-types of therapy to capture the processes that occurred in each session or patient encounter	Coordinated approach to therapy will reduce disability, improve quality of life, and may decrease psychological morbidity.						
	Offer visit to the ICU	May help with memories and adjustment to health status	Accompanied visit to ICU with GRA, medical staff, and family according to patient preference	ICU visit may help fill in gaps for some patients	High	High	GRA plus other staff according to patient preference	GRA familiar with individual patient history, and trained in common psychological morbidity and memories of ICU	ICU in which patient was cared for	Any time during acute hospital stay, or after hospital discharge if preferred
				May help with adjustment to illness or dealing with dreams and delusional memories	ICU visit optional					
				May reduce psychological issues	Timing to suit individual patient					
**Stage three – Hospital discharge planning**	Liaison with ward-based staff to ensure equipment and community referrals are in place before discharge home	Ensure services and equipment are in place during the transition from hospital to the community	Liaison between the GRA and other healthcare professionals to ensure services and equipment are in place at discharge	Ensure patient has correct services and equipment in place at home for discharge	Moderate	Moderate	GRA and other healthcare staff depending on patient needs	GRA familiar with patient history and social/home circumstance	General ward	Throughout ward stay to allow planning but in particular in the time leading up to discharge from hospital
				Ensuring patient is as supported at possible at the time of discharge from hospital						
**Stage four – Post-hospital discharge**	Provide contact details for GRA	A single point of contact to coordinate help if patient not coping in the community	Provide mobile phone number and advice to contact if required	Many patients are discharged home with significant disability	Moderate	Moderate	GRA	GRA familiar with individual patient case history	Community	Following hospital discharge at discretion of GRA
	Telephone patient at least once following discharge		A topic guide to ensure all issues are covered	Patients and families often uncertain where to turn for help	All patients and families will receive contact details			Trained to mobilise relevant hospital and community teams as required		Unsolicited contact within 1 week of discharge
			Ensure any equipment and community referrals are in place	Single point of contact to individual who knows their history well will enable rapid identification of problems and solutions	All patients will receive one unsolicited contact					Ongoing contact available until primary outcome measurement
				This will reduce patient/family stress, decrease chance of emergency readmission, and improve efficiency of use of community rehabilitation teams	Numbers of subsequent contacts determined by patient and family					
	GP discharge summary (example proforma included in Additional file 1)	A discharge summary completed by the GRA to provide additional information to GPs about the impact of the critical illness on the patient	A summary of functional ability across physiotherapy, occupational therapy, dietetics and speech and language therapy	GPs often only manage 1 to 2 patients a year that suffer a critical illness	High	Low	GRA	GRA familiar with the individual patient case history and status at the time of discharge	Office-based activity	Immediately following hospital discharge
			A short summary of psychological function	This information will increase their knowledge about the specific issues faced by the individual patient after a critical illness	All discharge summary letters will be completed with patient specific detail					
			A summary of community referrals made	The additional general information about common sequelae after critical illness will increase the GPs general knowledge of the issues faced by patients after critical illness and facilitate the identification of any issues that arise after discharge and can be managed by the GP	All summaries will include standard information about the general sequelae after critical illness					
			Information about typical physical and psychological sequelae after critical illness							

^1^Topic guides are available in Additional file 1. ^2^Proforma used to guide lay summary available in Additional file 1. ^3^Manual used was provided by the team that undertook the randomised controlled trial [20] and is in use in the National Health Service following the National Institute for Health and Care Excellence 83 recommendations [36]. ^4^The screening tools used are available in Additional file 1. ADLs, activities of daily living; GRA, generic rehabilitation assistant; GP, general practitioner.

## Discussion

We have provided a detailed account of the development of a complex rehabilitation intervention during the first 3 months following ICU discharge. In reflecting on this process, it is useful to consider whether the questions set out in the MRC complex intervention guidance were addressed during the construct development [27].

Interventions to improve recovery from critical illness could be developed to improve specific impairments (for example, leg muscle strength), specific functions (for example, ability to carry out ADLs), or specific symptoms (for example, fatigue). In practice, effective interventions are likely to affect multiple aspects of disability, including structure, function, activity, and participation domains [55], and may interact differently between patients. The intervention we describe will change care through a combination of new service development, specifically trained GRAs, and enhanced coordinated therapy across multiple rehabilitation domains. A methodological advantage of this model will be a reduced chance of bias and cross-over effects when comparing data to the parallel usual care group in the trial. In response to the perceived need for information a clear strategy to provide this will be included. We have therefore developed an intervention strategy with clearly defined components, timing, and a delivery model to implement the change.

We drew upon existing literature, a NICE guideline [36], qualitative work [31], and local data [32,33] to develop our construct and the rationale underpinning each component. Importantly, our construct was developed over a 5-year period with multiple episodes of iterative revision based on experience, internal and external review, and input from a wide range of relevant content experts and methodologists from different clinical and academic perspectives. We also engaged with patients and their families. We therefore believe that the final intervention is well supported by relevant evidence, and the plausibility of it translating into improved physical recovery, improvements in a range of other secondary outcomes, and an improved patient experience is high.

We have exerted significant effort to describe what we intend to do and a taxonomy to record and describe what actually happens in our trial. In referring heavily to the extended CONSORT guidance for reporting trials of non-pharmacologic interventions [28], we believe we will be able to address most checklist items when we report our findings. Specifically, as recommended for rehabilitation trials, we will describe numbers and timing of sessions, data on individual components of the intervention, the overall duration of different treatments, and when treatments will be tailored to individuals. The effort and resource required to provide this level of description during the trial is very substantial, but should enable detailed assessment of the key treatments that contribute to any effects observed. This detailed process evaluation will enable others to understand and implement the intervention if it is clinically effective. Importantly, it will also allow a clear description of the level of enhanced therapy that was not effective if results are negative, and clearly describe the usual care therapy that generated similar outcomes. This will be important for comparison with previous and future trials.

Existing evidence clearly indicates the need for novel approaches to rehabilitate patients after critical illness, because current disability levels are high in both physical and mental health domains [1,3,4,35]. A small randomised controlled trial published during development of our trial found improved functional outcomes in medical patients in whom an early mobilisation strategy was implemented during ICU care, compared with a usual care group receiving very limited mobilisation [49]. Recent systematic reviews of all published trials suggest mobilisation is important for recovery, although the quality of evidence is low [17-19]. These studies provide strong plausibility that an intervention coordinating enhanced physical, dietetic, and other rehabilitation will improve patient outcomes over the 3-month period following ICU discharge. We recently conducted a systematic review of costs following critical illness, but found few cost-effectiveness studies, high levels of heterogeneity, and wide variability in cost [16]. Despite variability, overall direct healthcare costs are high as the main determinant of cost is inpatient hospital stay. Analysis of potential cost-effectiveness of post-critical illness rehabilitation undertaken as part of the NICE guideline 83 [36] suggested that clinically effective interventions are very likely to be cost-effective [56]. Our trial includes a full health economic evaluation over 12 months follow-up.

Our feasibility study and pre-trial work indicated that the intervention can be implemented in the NHS institutions in which the trial is ongoing (the two major adult hospitals within NHS Lothian, Scotland) [32,33]. We believe the magnitude of the problem (70,000 to 100,000 surviving patients annually in the UK ), the strong recommendations of NICE guideline 83 [36], likely cost-effectiveness [56], and pressures on the acute healthcare sector mean widespread implementation of our complex intervention is likely if proved clinically and/or cost effective. We further believe that it will be widely practicable based on the information we have provided.

There are weaknesses to the methods used to develop our intervention. We did not undertake formal systematic reviews for the different components, although the incorporation of data from the NICE guideline development process reduced the risk of missing important evidence. A formal review of literature from other clinical settings (for example, the stroke or pulmonary rehabilitation literature) could have provided additional ideas for inclusion. We developed an intervention focussed on perceived needs of patients managed in the local healthcare setting, namely the UK NHS. The emphasis placed on locally acquired data could have introduced a researcher bias, although we attempted to minimise this through stakeholder involvement, and regular internal and external review. It is possible that our intervention may not address key needs in other healthcare systems, or may be addressing issues that are not relevant to those settings. This is difficult to avoid in complex intervention trials, where context is important, but justifies the need for careful description of intervention content and process. We did not use a pre-defined strategy for development, in part because the optimum methodology in this clinical setting is uncertain. MRC guidance [27] emphasises the importance of a cyclical iterative process when developing complex interventions. The 5-year cycle we used prior to embarking on a trial enabled modelling and piloting, but made the incorporation of emerging new data challenging. Ultimately, intervention development ended when several cycles of grant submissions resulted in a successful funding award. Several potential interventions were not included. These included controlled strategies during the period of mechanical ventilation in ICU, where interventions to increase mobilisation or maintain muscle activity show promise as strategies to decrease long-term functional disability [17-19]. Similarly, a formal exercise programme following hospital discharge was not included, although this was not effective in a recent trial [22]. Other issues that could have been addressed included management of delirium, sleep, and early traumatic memories. Our trial will evaluate the effectiveness of enhanced multidisciplinary rehabilitation focussed mainly on the acute hospital stay following ICU discharge. It may be difficult to exclude effect modification from other interventions that were not included or controlled. Despite this, both positive and negative trial results will inform future clinical practice and research design. In developing our intervention, a challenge was balancing the potential benefits of a more complex design over a longer time frame with the risk of greater protocol non-compliance, recruitment fatigue, and reduced capability to describe process in detail. These issues are challenging in this research field, but we believe they highlight the importance of clearly describing what was done in individual trials. A recent trial of enhanced physiotherapy following ICU admission had a longer term intervention during several stages of patient recovery, but failed to achieve adequate power as a result of low recruitment rates [23].

## Conclusions

The development, implementation, and evaluation of complex interventions to promote recovery following critical illness are challenging. This detailed description of the intervention used in our trial using the principles set out in the MRC complex intervention framework [27] provide important additional information to the published protocol. This should help readers understand the trial results, maximise the external validity of the data, and help others implement the intervention, if proved clinically and cost-effective. Our detailed account of construct development and description provide a model for others undertaking similar complex intervention trials.

## Abbreviations

ADLs: activities of daily living; GDG: guideline development group; GRA: generic rehabilitation assistant; HRQoL: health-related quality of life; MRC: Medical Research Council; NHS: National Health Service; NICE: National Institute for Health and Care Excellence.

## Competing interests

The authors declare that they have no competing interests.

## Authors’ contributions

TSW, LGS, JLM, PR and GH conceived the study and completed pilot and feasibility work. Specialist input to the study design and protocol development was provided by TSW, SJB and SJM (critical care perspective), LGS (physiotherapy/rehabilitation), PR and JER (nursing), JLM (dietetics), AMH (Psychiatrist in Psychotherapy), JFF (health economics), and GDM (study design and statistics). Advice on health service reorganisation aspects was provided by GH and SJM. All authors (with the exception of SJB) were involved in the acquisition of funding. All authors contributed to the drafting and revision of the paper. All authors read and approved the final manuscript.

## Supplementary Material

Additional file 1 This document provides supplementary information to help increase the understanding of the context and content of the intervention delivered during the RECOVER trial. Click here for file
