# Supplementary material for: A rehabilitation intervention to promote physical recovery following intensive care: a detailed description of construct development, rationale and content together with proposed taxonomy to capture processes in a randomised controlled trial
Source: Trials. 2014 Jan 29;15:38. doi: 10.1186/1745-6215-15-38 (PMC4016544; doi:10.1186/1745-6215-15-38)
Supplement: Additional file 1 — This document provides supplementary information to help increase the understanding of the context and content of the intervention delivered during the RECOVER trial. [file 1745-6215-15-38-S1.doc]

**Supplementary Material**

This document provides supplementary information to help increase the understanding of the context and content of the intervention delivered during the RECOVER trial.

Contents:

1. Summary of the GRA training programme p1
2. Triggers to facilitate referral to different allied health disciplines p3
3. Patient-centred goal setting sheet p7
4. Physical and psychological guides used during the consultant visit p8
5. An example of a lay summary p15
6. The discharge sheet completed after hospital discharge p18

Summary of the GRA training programme

The table below provides detail about the training programme and competencies completed by the GRA’s.

| General background to intensive care and the management of critical illness | - The history of critical care, critical care in Scotland: some facts and figures, the “typical” ICU patient and the critical illness journey? - Critical illness: the patient’s perspective including the ICU experience; ward transfer/ “relocation stress”; experiences of ward-based care; experiences of ward-based rehab; experiences of hospital discharge; life at home - Visit to the intensive care unit - Review of intensive care medical notes |
| --- | --- |
| Background to the study | - Overview of ward-based rehabilitation and the study protocol - Introduction to the discipline specific triggers and referral processes - Introduction to the manual |
| Physical problems  after intensive care | - Aetiology, prevalence and experiences of common physical problems following intensive care including muscle wasting; weakness; fatigue; weight loss; joint pain/stiffness; breathlessness; weight loss; loss of appetite; taste changes; swallowing problems. |
| Psychological  problems after  intensive care | - Aetiology, prevalence and experiences of psychological problems following intensive care including amnesia; delirium/false memories; post-traumatic stress disorder; anxiety; depression; cognition. |
| Psychological  training | - Administration of psychological screening tools - Introduction to self-help strategies and resources - Normalisation |
| Patient Centred Goal  Setting | - Background to patient-centred goal setting - Principles and practice in patient-centred goal setting |
| Generic skills | - Completion of generic based competencies including communication; the ability to respect and value people; completion of documentation; effective and efficient management of caseload; promoting rehabilitation after critical illness; safe manual handling and the ability to implement pacing and energy conservation techniques. |
| Dietetic Training | - Shadowing of Dietetic Staff and Dietetic Assistants - Shadowing of the catering service including a kitchen visit - Supplement tasting - Completion of dietetic based competencies including an understanding of the theory relating to malnutrition; an awareness of different techniques of nutritional assessment; ability to accurately assess nutritional intake; ability to undertake a nutritional review; understanding of the different interventions to increase nutritional intake; working knowledge of enteral feeding pumps. |
| Occupational  Therapy Training | - Shadowing of Occupational Therapy Staff and Occupational Therapy Assistants - Training in systems to allow equipment ordering and setting up of basic home care. - Completion of occupational therapy based competencies including the ability to carry out education and safe fitting of equipment; ability to carry out basic components of an occupational therapy assessment; practice of techniques in a care plan with a knowledge of issues that may affect practice including motor, sensory, cognitive and general issues; completion of an environmental visit; assist with a home visit; awareness of techniques used with different patient groups. |
| Physiotherapy  Training | - Shadowing of Physiotherapy Staff and Physiotherapy Assistants - Shadowing of pulmonary rehabilitation - Completion of physiotherapy based competencies including awareness and safe use of different walking aids; mobility practice with appropriate aids and gait pattern; awareness of safety issues around mobilisation; correct and safe stair practice; basic theory underpinning exercise; basic knowledge of musculoskeletal structures and normal movement patterns; ability to carry out unsupervised treatment sessions; ability to carry out TENS, hot/cold packs and cryocuff. |
| Speech and  Language Therapy  Training | - Shadowing of speech and language therapy Staff and speech and language therapy Assistants - Completion of speech and language therapy based competencies including understanding of the normal swallow; understanding of dysphagia and associated risks; basic understanding of a speech and language therapy dysphagia assessment and management; understanding of referral routes; ability to identify different types of communication difficulty. |
| Other suggested  resources | http://www.ics.ac.uk/about_us/critpal  http://www.healthtalkonline.org  http://www.i-canuk.com  http://www.icusteps.com |

Trigger tools to facilitate referral to different allied health disciplines

The following trigger forms were used to facilitate referral for dietetics, occupational therapy, physiotherapy and speech and language therapy as required.

**Dietetic Trigger Form**

To be completed by the generic rehabilitation assistant for participants in the intervention group at admission to the ward and on a weekly basis.

| Date of completion | D | D | M | M | M | Y | Y | Y | Y |
| --- | --- | --- | --- | --- | --- | --- | --- | --- | --- |

|  | YES | NO |
| --- | --- | --- |
| 1. Is the patient currently being seen by a Dietitian? |  |  |
| **If the answer to question 1 is YES liaise with the Dietitian for a treatment plan.** If the answer to question 1 is NO or is unknown please continue | | |
| 2. Is the patient currently receiving artificial feeding e.g. Enteral/parenteral nutrition |  |  |
| 3. Does the patient have a MUST score of ≥ 2 |  |  |
| 4. Is the patient reporting difficulties with eating e.g. evidence of poor food intake consistently for greater than 48 hours? |  |  |
| *If the answer to any of questions 2, 3 and/or 4 is YES please speak with the ward dietitian* | | |

**Occupational Therapy Trigger Form**

| Date of completion | D | D | M | M | M | Y | Y | Y | Y |
| --- | --- | --- | --- | --- | --- | --- | --- | --- | --- |

|  | YES | NO |
| --- | --- | --- |
| 1. Is the patient currently being seen by an Occupational Therapist at present? |  |  |
| **If the answer to question 1 is YES liaise with the Occupational Therapist for a treatment plan.** If the answer to question 1 is NO or is unknown please continue | | |
| 2. Is the patient independent in transfers (bed chair; bath; shower; toilet) |  |  |
| 3. Is the patient able to wash and dress independently |  |  |
| *If the answer to both questions 2 and 3 is YES the patient is unlikely to require a referral to Occupational Therapy* *If the answer to either questions 2 & 3 is NO go to question 4* |  |  |
| 4. Is the patient able to mobilise with a zimmer frame and assistance of one? |  |  |
| *If the answer to question 4 is YES complete a front sheet and background information and liaise with OT* ***If the answer to question 4 is NO continue to monitor patient until they are able to mobilise with zimmer frame and assistance of one.*** |  |  |

**Physiotherapy Trigger Form**

| Date of completion | D | D | M | M | M | Y | Y | Y | Y |
| --- | --- | --- | --- | --- | --- | --- | --- | --- | --- |

|  | **YES** | **NO** |
| --- | --- | --- |
| 1. Is the patient currently being seen by a physiotherapist at present |  |  |
| **If the answer to question 1 is YES liaise with physiotherapist for treatment plan.**  **If the answer to question 2 is NO or is unknown answer question 2** | | |
| 2. What is the patients’ **NORMAL** level of mobility? (please cross)  **Aid?**  Independent (No Aid) Delta Frame  Walking Stick x1 Trolley  Walking Stick x 2 Gutter Frame  Crutches Hoist (Stand Aid)  Zimmer Frame Hoist (Maxihoist)  Wheelchair Bedbound Assistance? Supervision Assistance of 2  Assistance of 1  **Other?** Please Specify _____________  2a What is the patients’ **CURRENT** level of mobility? (Use wording from Qu. 2)  **…………………………………………………………………………………………….**  2b Is this patients’ **CURRENT** level of mobility different from **NORMAL?** No Yes |  |  |
| **If the answer to question 2b is NO it unlikely the patient requires a referral to physiotherapy.**  **If the answer to question 2b is YES liaise with the ward physiotherapist.** |  |  |

**Speech and Language Therapy Trigger Form**

| Date of completion | D | D | M | M | M | Y | Y | Y | Y |
| --- | --- | --- | --- | --- | --- | --- | --- | --- | --- |

|  | **YES** | **NO** |
| --- | --- | --- |
| 1. Is the patient currently being seen by a speech and language therapist? |  |  |
| **If the answer to question 1 is YES liaise with the speech and language therapist for a treatment plan.**  **If the answer to question 1 is NO or is unknown answer the following questions** | | |
| 2. Does the patient present will any of the symptoms below? | **YES** | **NO** |
| **General** | | |
| Tracheostomy still insitu |  |  |
| Decannulation within 48 hours |  |  |
| **Communication** | | |
| Slurred or stumbling speech |  |  |
| Weak voice, altered voice quality (harsh; husky; nasal) |  |  |
| Unable to produce normal sentence structure (e.g. omitting nouns/verbs) |  |  |
| Word finding difficulty |  |  |
| Difficulty understanding (not related to hearing/confusion) |  |  |
| Unusual rhythm/lack of speech |  |  |
| **Swallowing** | | |
| Wet-gurgly voice |  |  |
| Feeling of food sticking in throat |  |  |
| Difficulty chewing |  |  |
| Food residue in mouth after swallowing |  |  |
| Losing food/fluid from mouth |  |  |
| Taking a long time to initiate swallow |  |  |
| Breathlessness when eating |  |  |
| **If any of the above symptoms are present please liaise with the Speech and Language Therapist.** | | |

Patient-centred goal setting sheet

This patient-centred goal-setting sheet provided a framework for identifying patient-centred rehabilitation goals

**Patient Centred Goal Setting Record**

**RECOVER: Rehabilitation After Critical Illness**

**Patient’s Name**: **D.O.B.**  **Key Worker:**

**Patient Priorities:**

| **Date** | **Current Status** | **Goal** | **Outcome** | **Review Date** |
| --- | --- | --- | --- | --- |
|  |  |  |  |  |
|  |  |  |  |  |
|  |  |  |  |  |
|  |  |  |  |  |
|  |  |  |  |  |

**Outcome Codes:**  A = Achieved P = Partially Achieved E = Exceeded N = Not Achieved

Reasons for goals not being achieved: -

Physical and psychological guides used during the consultant visit

RECOVER: Consultant checklist (physical)

| **Issues** | **Example questions/prompts** | **Explanation/reassurances** | **Action/suggestions** |
| --- | --- | --- | --- |
| **Generalised weakness** | - It’s very common for patients to suffer from overall weakness after Intensive Care. | - How severely people are affected seems to depend on things like how fit people were before their illness and how long they spent in Intensive Care. - Patients who spend a long time in Intensive Care seem to suffer the most severe forms and can take a long time to recover, whereas people who’ve spent a short while in ICU seem to “bounce back” relatively quickly | - You will be receiving quite intensive physiotherapy to help get you back on your feet. - The booklet we’ve given you will also give you some exercises to do by yourself when you’re ready. - Remember that it might take you quite some time to get back to normal. |
| **Muscle**  **wasting** | - Patients often lose a lot of muscle whilst in Intensive Care. Have you noticed any changes in your arms or legs? | - Patients can lose up to 2% of their muscle for every day they spend in ICU. - The legs are often the worst affected. - How severely people are affected seems to depend on things like how fit they were before their illness and how long they spend in Intensive Care. | - As above - Physiotherapy will help you to regain the muscle you’ve lost. - The booklet we will give you some exercises to do by yourself when you’re ready. - You’ll also need to make sure that you’re getting enough of the right foods to build yourself up. The dietitian will help with that. |
| **Fatigue** | - Have you been feeling more tired than normal since you came out of Intensive Care? | - Feeling very tired is really common, probably as a combination of things like the muscle wasting, sleep disturbance and as a basic response to how ill you’ve been. | - The Rehab. Assistant will help you with ways to deal with tiredness (e.g. pacing yourself). - It’s important to try to learn these skills before you go home as the tiredness can take months to improve. |
| **Joint**  **stiffness** | - Any problems with stiff or painful joints? | - Some people suffer from this after Intensive Care (particularly the shoulders and knees), probably due to being in bed for a long time. | - The stiffness should ease off as you start to become more mobile - The booklet we’ve given you will give advice on exercises to do to help with this - Some people, however, continue to have problems with stiff and painful joints. If this persists after you go home, speak to your GP |
| **Poor mobility** | - How are you on your feet compared to how you were before? | - People often suffer from poor mobility, often due to a combination of things like the muscle wasting, tiredness, poor balance, stiffness and joint pain. | - The Rehab Assistant will help you practice with things like getting in and out of bed and walking, until you are able to do them by yourself. - The exercises we will give you will help you get back on your feet |
| **Breathlessness** | - Have you noticed any changes in your breathing? - Do you get out of breath more quickly than before? | - After being in bed for a number of days, your ability to exercise can be markedly reduced - Even a simple activity like getting out of bed can make you feel breathless as your body is having to work hard | - The Rehab Assistant will help you with ways to build up your stamina - The booklet will also give you advice on this - If you gradually increase your activity levels, the breathlessness should become less of a problem |

RECOVER: Consultant checklist (physical)

| **Issues** | **Example questions/prompts** | **Explanation/reassurances** | **Action/suggestions** |
| --- | --- | --- | --- |
| **Weight loss** | Have you lost (a lot of) weight since your illness? | - Weight loss is very common, even though we’re very careful about feeding patients while they’re in Intensive Care…either through a tube in the nose or directly into a vein. - Patients can lose up to 20% of their body weight while in Intensive Care | - Eating well will help you regain the weight you’ve lost, but it can sometimes take a long time to put it back on |
| Appetite | How’s your appetite been? Are you eating ok? | - A poor appetite is very common after Intensive Care. | - Ask your relatives to bring in some of the things you really like (the staff won’t be allowed to store or heat up things like cooked food, though). - You might not actually feel like it though, when it’s in front of you! - If your appetite’s not as good as it was, it might help to have smaller portions, just until your appetite comes back. - Try to eat small but frequent meals. - If the dietitian has recommended high protein drinks or extra snacks, remind the staff to bring them to you. |
|  | Have you just been too tired to eat? |  | - Sometimes it can feel like bit of an effort, but it’s important to try to eat. - Ask the staff if you need help to eat or if you need help to sit up. |
|  | Do the portion sizes put you off? |  | - Again, try to eat small but frequent meals. |
| Impaired swallow | Are you having any difficulty swallowing? | - People sometimes have problems swallowing if they’ve needed the breathing tube for a long time. - This is often temporary. | - The Rehab Assistant will keep an eye on this and will either refer you for specialist help (SLT) or will help you with swallowing exercises - If your swallow is very poor, it’s sometimes necessary to feed you through a tube in the nose (or directly into a vein) until your swallowing improves |
| Taste changes | Does your food taste ok? | - People often find that their food is tasteless…or that it tastes different e.g. sweeter or saltier than normal, or that it has a metallic taste. - This is often temporary and will get better over time | - It’s still important to persevere with eating - Sometimes strongly flavoured food is more palatable - Remember that your sense of taste will come back |

RECOVER: Consultant checklist (physical)

| **Issues** | **Example questions/prompts** | **Explanation/reassurances** | **Action/suggestions** |
| --- | --- | --- | --- |
| **Skin changes** | Have you noticed that your skin has been particularly dry or itchy since you came out of Intensive Care? | - This is very common after Intensive Care and is almost always temporary |  |
| Scarring | You may have noticed a number of red or raised areas | - These tend to be around the neck, wrist or groin area where various lines have been…or you may have needed a tracheostomy | - Some people find them upsetting, seeing them as a reminder of what has happened… while others see them as “battle scars” - The marking around the throat area (where you had a tracheostomy) can be particularly embarrassing for some - The scarring will tend to fade over time. - If the scarring doesn’t fade over a number of months and is particularly upsetting to you, speak to your GP |
| **Nail changes** | Have you noticed any changes in your nails? | - The nails can often become dry and brittle, and can develop ridges | - Again, this is temporary. |
| **Hair loss** | Have you noticed any changes in your hair? | - Thinning or loss of hair is very common after Intensive Care | - Although this can be sometimes be very embarrassing, this is usually temporary, resolving over a matter of months. |
| **Voice changes** | Have you noticed any changes in your voice? | - Some people notice a change in their voice, usually volume and tone - This is common among patients who have a breathing tube in place for longer periods of time, and among those who have had a tracheostomy | - As with most of the issues we’ve already discussed, this is often temporary. - If this doesn’t get better over a number of months, you should go and see your GP |

**RECOVER: Consultant checklist (psychological)**

| **Psychological**  **Psychological** | **Issues** | **Example questions** | **Prompts/probes** | **Explanation/reassurances** | **Action** |
| --- | --- | --- | --- | --- | --- |
|  | Amnesia | What can you remember about your time on Intensive Care?  Is there anything niggling you that you simply can’t remember…or can only remember bits and pieces? | It’s very common that people don’t remember very much…  What you remember might not make much sense…  Does it bother you that you can’t remember very much? | This is probably due to a combination of the drugs we give you on ICU to keep you sleepy and comfortable, and how ill you were.  People are often able to “cobble together” what happened to them by asking family members, speaking to the staff from Intensive Care, or even visiting the Intensive Care Unit.  Some people like to think of their “not remembering” as a good thing, as a sort of protective mechanism, while others desperately want to know what happened to them. Neither is right or wrong; these things just vary between people. | I have a summary here of what happened to you, which you can keep. Would you like to go through it now, or would you rather leave it for another time?  Some people find that visiting the ICU helps “jog the memory” in a helpful way. Some people would rather not. Either way is entirely up to you. If you think it might help you, we could arrange this for you when you’re ready. |
|  | **Issues**  Delirium/PTSD | **Example questions**  Did you have any strange dreams or experiences while you were in Intensive Care? | **Prompts/probes**  It’s very common for people to have these whilst in ICU…  The dreams or hallucinations that people have can seem very real…  Have you found these memories upsetting? | **Explanation/reassurances**  Again, these are probably due to a combination of the drugs we give you on Intensive Care to keep you asleep and comfortable, and how ill you were.  The dreams that people have can often seem very bizarre or complex. Some people think of them as a way of “keeping the mind occupied” or the mind’s way of making sense of what’s going on.  For some people, these dreams seem to be bizarre misinterpretations of reality. They often include, for example, members of staff or friends and family. Patients on a pressure relieving mattress, for example, often believe they’ve been on a boat…  People often wonder why they might dream about this or dream about that. Many people find it very useful to speak to their relatives about these dreams and experiences, if only to check whether what they “remember” actually happened. Some find that difficult, though, as they’re worried that their relatives might think they’re going mad.  It’s also very common for people to have “memories” or dreams in which others are trying to harm or even kill them. We’ve found from talking to previous patients, that while these memories can be very vivid and upsetting, they generally tend to fade over time. | **Action**  Sometimes the dreams people have are actually nightmares. They can intrude on your everyday thoughts, interrupt your sleep or even make you anxious about going to sleep. If this is the case, we can either arrange for you to see someone while you’re still in hospital (liaison psychiatry), or we would suggest you see your GP after you get home.  Are you still having these sorts of thoughts and memories?  Some people can have upsetting intrusive thoughts about their experience or “flashbacks” where they feel they are reliving it (often referred to as post traumatic stress disorder) for some weeks or months after discharge home. If you continue to have these symptoms for more than 2 weeks, you should probably go and talk to your GP. |

| **Psychological** | **Issues** | **Example questions** | **Prompts/probes** | **Explanation/reassurances** | **Action** |
| --- | --- | --- | --- | --- | --- |
|  | Comprehension | What’s your understanding of how you ended up in Intensive Care? | What have you been able to piece together from what you remember and what your relatives have told you? | It’s often very difficult for patients to come to terms with how very ill they’ve been, because they’ve been “asleep” for the worst part of their stay in Intensive Care.  Often, people can’t remember the few days before they came into hospital…and sometimes the few days after transfer to the ward can be a bit hazy.  Sometimes people find it difficult to ask their relatives about what happened because they find it frightening or they don’t feel quite ready to hear about it. Others feel that their relatives have been through a lot, having had to see you so very ill in Intensive Care. | I have a summary here of what happened to you, which you can keep. Would you like to go through it now, or would you rather leave it for another time?  Some people find that visiting the ICU helps “jog the memory” in a helpful way. Some people would rather not. Either way is entirely up to you. If you think it might help you, we could arrange this for you when you’re ready. |
|  | Poor  concentration | Have you been finding it difficult to concentrate? | You might be finding it difficult, for example, to concentrate on reading a newspaper or a book at the moment. | This is also very common. | It may take several weeks or sometimes months to get back to normal. It’s just all part of the body’s recovery process. |
|  | Anxiety/depression | How do you feel about what’s happened to you? | Have you been feeling a bit anxious/wound up, or a bit down in the dumps about what’s happened? | It’s very common for people to feel like that after being in Intensive Care. | It might not always be easy, but it often helps to talk about how you’re feeling.  These feelings do tend to reduce over time but it may take some months. If they persist then you should see your GP. |

An example of a lay summary

**RECOVER – Example 1**

**Your time in Intensive Care**

**Why am I being given this sheet?**

Often, patients remember only “bits and pieces” of their time in Intensive Care. This is very common and is probably due to a combination of the drugs you received to keep you comfortable (“sedation”) and the nature of your illness. In order to help you understand what happened to you, one of our Intensive Care Consultants has put together the following information, after looking at your medical notes. Please ask him/her if you are unclear of anything or would like to know more. Your family and friends will also be able to help you “fill in the gaps”. Some people find a visit to the Intensive Care Unit useful. If you would like to do this before you go home, please ask and we will arrange this for you.

**How did I end up in Intensive Care?**

You were diagnosed in September last year with acute pancreatitis (inflammation of the pancreas gland) caused by gall stones. Since then you have had multiple hospital admissions with complications associated with this condition, particularly in relation to pseudo-cysts (these are fluid filled collections beside the pancreas gland). Your latest admission to hospital was in the middle of March, again with complications associated with the pseudo-cysts. On the 18^th^ March your condition deteriorated such that you were transferred to the Intensive Care Unit. This is because you had breathing difficulties due to a low oxygen level. You were given assistance with your breathing by wearing an oxygen hood and having fluid drained off from the left lung. This fluid collection beside the lung was connected to another fluid collection in your abdomen, arising from a pseudo-cyst. Your condition improved over the course of two days and you were transferred, initially to the High Dependency Unit and then the ward. However, on the 22^nd^ March you again developed problems with your breathing, warranting your return to the Intensive Care Unit. At that stage your breathing difficulties were thought to be due both to the fluid collection outside your lungs and a pneumonia developing in the lung. You remained in the Intensive Care Unit for five weeks, chiefly because of infection both in the lung and pancreas gland.

**What were the main problems while I was in Intensive Care?**

The main problems you experienced in Intensive Care were:-

1. Recurrent infections and collections in relation to the pancreas gland

The pseudo-cyst collections at times communicated with your chest.

1. ARDS – this is an inflammatory process that develops in the lung and results in low oxygen levels. This was due to your pancreatitis.
2. A hospital-acquired pneumonia involving MRSA
3. Gut failure. This was because of the inflammation and infection in your abdomen.
4. Malnutrition, due to poor dietary intake.
5. Delirium (confusion arising as a consequence of your illness and sedative drugs)

***To summarise your stay***

On your second admission to Intensive Care from the 22^nd^ March to the 23^rd^ April you had breathing difficulties caused by a pneumonia and fluid outside the lung compressing the lung itself. You were given antibiotics to treat this and drugs to support your blood pressure. Because your gut was not functioning adequately at this stage you also received drip feeding directly into the blood stream. At times you required drugs to treat an irregular heart beat but this improved with time.

Over the course of the next few weeks you received repeated scans of your abdomen to see how big the fluid collections were and if they warranted drainage by insertion of a tube drain. You were very weak and malnourished and your lungs were also very inflamed so to try and wean you off the breathing machine, a tracheostomy was performed on the 31^st^ March. This is a tube directly inserted into the windpipe through the neck. With repeated courses of antibiotics and ongoing drip feeding your condition slowly improved and you were gradually weaned from the ventilator onto simple oxygen tubing. Finally by around the 18^th^ April, you were well enough for the tracheostomy tube to be removed. However, you were still very weak and had evidence of an infection in your blood stream, presumably coming from the pancreas gland. You were kept in ICU for a further few days to ensure that this was being treated adequately.

**What kind of medical treatments did I have?**

1. Life support on a ventilator.
2. A tracheostomy
3. Drugs to support your blood pressure
4. Multiple courses of antibiotics
5. Multiple scans of your abdomen with insertion of drainage tubes
6. Nutrition through a drip

**What kind of routine treatments did I get?**

The Intensive Care doctors kept a very close eye on your condition, often reviewing your care several times a day. You were also seen by specialist clinicians due to the nature of your illness. You were looked after by highly trained nursing staff on a one to one basis for much of your stay. You were seen by a physiotherapist every day during your time on the Intensive Care Unit. They helped to keep your chest clear and carried out exercises on your arms and legs. You were also seen regularly by a dietician, and received nutrients either through a tube in your nose or a drip into a vein. Other staff included pharmacists and radiographers.

**How long was I on “life support” (also known as a “breathing machine” or “ventilator”) for?**

You were placed on a breathing machine at the start of your second, prolonged, ICU stay on the 22^nd^ March. A tracheostomy was performed on the 31^st^ March to help get you off the ventilator and by the 18^th^ April you had managed to cope for 24 hours off the life support machine and thus the tracheostomy tube was removed.

**How long was I in Intensive Care for?**

You had two admissions to Intensive Care, the first one from the 18^th^ to the 20^th^ March and the second one from the 22^nd^ March to the 23^rd^ April, a total of 3 and 33 days.

**What kind of problems am I likely to have after Intensive Care**

A number of general problems have been associated with spending time in Intensive Care. We have given you a booklet (the “Intensive Care Recovery Manual”) which will provide you with more information about these problems, how to deal with these yourself and when to seek professional advice. We will also write to your GP to remind him/her of the problems that Intensive Care patients sometimes have.

In addition to these general problems which most people have after Intensive Care you could get other specific complications related to what caused your critical illness i.e. acute pancreatitis. Hopefully you will not have any further infections in the gland but it can cause some ongoing discomfort. A few people have a tendency to have a form of diabetes since the pancreas gland produces insulin. Also, you were very malnourished by the end of your critical care stay and it may take some time for your strength and weight to return to normal. Also since it was felt that gall stones were the cause of your original pancreatitis you may need to have your gall bladder removed once you have made a full recovery.

**How long will it take to get back to normal?**

The length of time it takes to recover can vary enormously between people. We have given you a booklet (the “Intensive Care Recovery Manual”) which will provide you with more information about the ways in which you can help the recovery process along. If you would like more advice after you go home, you can also telephone the Rehab Assistant (between normal office hours, Monday to Friday) on the following numbers.

The discharge sheet completed after hospital discharge


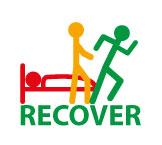


Address

The RECOVER study: Evaluation of a **Re**habilitation **Co**mplex Intervention for patients following Intensi**ve** Care Discha**r**ge.

Dear

Re:

You will recently have received a letter informing you of the participation of the above patient in the RECOVER study (a randomised controlled trial of enhanced physical and nutritional rehabilitation among survivors of critical illness following discharge to the general ward). Please find enclosed a discharge summary sheet for the patient. Information about typical (sometimes late) morbidity associated with critical illness has also been included. If you require any additional information, please contact XXXXXXXXX on XXXXXXXXXX (Assistant Practitioner on the RECOVER Study).

Yours sincerely,


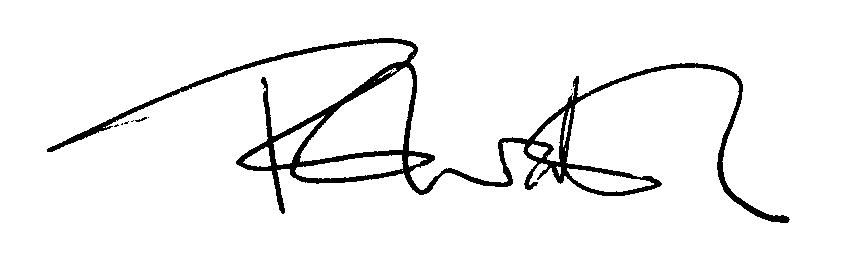


Professor Tim Walsh (Principal Investigator)


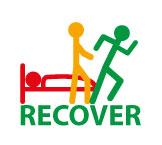


| **Discharge destination** | Home / other hospital / family / other: |
| --- | --- |
| **Functional Ability** | |
| **Physiotherapy** | Mobility:  Transfers (bed/chair):  Balance:  Stairs: |
| **Occupational Therapy** | Personal Activities of Daily Living (washing / showering etc):  Domestic Activities of Daily Living (laundry / shopping etc):  Equipment: |
| **Dietetic** | Eternal feeding / supplements / altered diet etc: |
| **Speech & Language** |  |
| **Community Referrals** | Social Work / Enablement / Community PT/OT/Dietetics/SALT etc |
| **Any additional information:** | |
| **Psychological function*** | |
| Social support | Lives with spouse/partner/relative □ Lives with friend □  Lives alone □ |
| *Participants in the RECOVER study are not formally tested for psychological morbidity (anxiety, depression and PTSD), although this is prevalent among ICU patients. Please see below for recommended assessment tools. | |


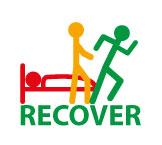


**Typical (sometimes late) sequelae of critical illness**

| **Physical** | Muscle wasting | Occurs most commonly in the extremities.  Patients may lose ≤2% muscle mass for each day spent in ICU |
| --- | --- | --- |
|  | Joint stiffness | Occurs most commonly in the shoulders and knees. Generally resolves with increasing activity. |
|  | Fatigue | Almost universal and often slow to resolve. |
|  | Generalised weakness | Often slow to resolve. |
|  | Impaired exercise tolerance | Due to deconditioning. Often slow to resolve. |
|  | Weight loss | Often due to reduced appetite, impaired swallow or taste changes in food.  Patients may lose ≤20% of their admission body weight |
|  | Voice changes  (e.g. hoarseness) | Often associated with prolonged intubation and usually temporary.  N.b tracheal stenosis is a late complication, occurring in between 10 and 20% of intubated patients. |
|  | Dry, itchy skin |  |
|  | Thinning of the hair or temporary hair loss |  |
| **Psychological** | Anxiety1 | Occurs in 12-43% of patients |
|  | Depression1 | Occurs in 10-30% of patients |
|  | Post Traumatic Stress symptomatology2 | Characterised by avoidance behaviours, hyperarousal and distressing and intrusive “flashbacks”.  Occurs in 5-65% of patients |
|  | Cognitive dysfunction | Characterised by deficits in attention/concentration, executive function (planning/organising) and memory. Occurs in ≤33% of ventilated patients |
|  | Sleep disturbance |  |

1. The HADS is recommended for the assessment of ICU survivors

2. The Impact of Events scale or Davidson’s Trauma Scale are recommended for the assessment of ICU survivors
